# Mycorrhizal Switching and the Role of Fungal Abundance in Seed Germination in a Fully Mycoheterotrophic Orchid, *Gastrodia confusoides*

**DOI:** 10.3389/fpls.2021.775290

**Published:** 2022-01-13

**Authors:** Yuan-Yuan Li, Margaux Boeraeve, Yu-Hsiu Cho, Hans Jacquemyn, Yung-I Lee

**Affiliations:** ^1^Beijing Key Laboratory of Seed Disease Testing and Control, College of Plant Protection, China Agricultural University, Beijing, China; ^2^Department of Biology, Plant Conservation and Population Biology, KU Leuven, Leuven, Belgium; ^3^Biology Department, National Museum of Natural Science, Taichung, Taiwan; ^4^Department of Life Science, National Taiwan University, Taipei, Taiwan

**Keywords:** *Gastrodia*, mycoheterotrophy, mycorrhiza, orchid, saprotrophic fungi, seed germination

## Abstract

Mycorrhizal associations are essential for orchid germination and seedling establishment, and thus may constrain the distribution and abundance of orchids under natural conditions. Previous studies have shown that germination and seedling establishment in several orchids often decline with increasing distance from adult plants, resulting in non-random spatial patterns of seedling establishment. In contrast, individuals of the fully mycoheterotrophic orchid *Gastrodia confusoides* often tend to have random aboveground spatial patterns of distribution within bamboo forests. Since *G. confusoides* is parasitic on litter-decaying fungi, its random spatial patterns of distribution may be due to highly scattered patterns of litter-decaying fungi within bamboo forests. To test this hypothesis, we first identified the main mycorrhizal fungi associating with developing seeds and adult plants at a bamboo forest site in Taiwan using Miseq high-throughput DNA sequencing. Next, we combined seed germination experiments with quantitative PCR (qPCR) analyses to investigate to what extent the abundance of mycorrhizal fungi affected spatial patterns of seed germination. Our results show that seed germination and subsequent growth to an adult stage in *G. confusoides* required a distinct switch in mycorrhizal partners, in which protocorms associated with a single *Mycena* OTU, while adults mainly associated with an OTU from the genus *Gymnopus*. A strong, positive relationship was observed between germination and *Mycena* abundance in the litter, but not between germination and *Gymnopus* abundance. Fungal abundance was not significantly related to the distance from the adult plants, and consequently germination was also not significantly related to the distance from adult plants. Our results provide the first evidence that the abundance of litter-decaying fungi varies randomly within the bamboo forest and independently from *G. confusoides* adults.

## Introduction

Both dispersal and recruitment limitation represent major factors that affect the distribution and abundance of plant populations (Eriksson and Ehrlén, 1992; Clark et al., 2007). Dispersal of seeds into favorable microsites is a first critical step shaping recruitment and distribution patterns of plants (Condit et al., 1992; Nathan and Muller-Landau, 2000; Wang and Smith, 2002; Getzin et al., 2014). When seeds fail to reach suitable habitats, no recruitment will occur at these sites and plants are considered dispersal or seed limited (Clark et al., 2007; Poulsen et al., 2007). If, on the other hand, seeds are widely dispersed, but fail to recruit because habitats are unsuitable, plants are considered habitat- or microsite-limited (Münzbergová and Herben, 2005; Johansson and Ove Eriksson, 2013). Most seed introduction and addition experiments have shown that supplemental addition of seeds to populations almost always results in increased seedling recruitment, indicating that the majority of plant populations are to some extent seed limited (Clark et al., 2007; Poulsen et al., 2007).

For plant species that critically depend on mycorrhizal symbiosis for seed germination and seedling establishment, the availability of appropriate mycorrhizal fungi may represent an influential portion of favorable microsite, and this is particularly true for orchids (Diez, 2007; Jacquemyn et al., 2007; McCormick and Jacquemyn, 2014; Waud et al., 2016; McCormick et al., 2018). Orchids have dust-like seeds that consist of rudimentary embryos without endosperm at the time of seed dispersal (Arditti and Ghani, 2000). Under natural conditions, the germination of orchid seeds and the early development of protocorms rely on mycorrhizal associations for nutrient supplies (Smith and Read, 2008; Rasmussen and Rasmussen, 2009; Rasmussen et al., 2015). Therefore, the availability of suitable mycorrhizal fungi is considered to be a limiting factor in seedling recruitment that significantly affects the abundance and spatial distribution of orchids (Rasmussen and Whigham, 1998; McKendrick et al., 2002; McCormick and Jacquemyn, 2014; McCormick et al., 2018).

Because orchid mycorrhizal (OrM) fungi can grow well without the association with orchids, it is generally assumed that OrM fungi are distributed independently from orchids and are therefore more widespread than the distribution of orchids suggests (McCormick et al., 2018). Previous studies using seed introduction experiments have indeed shown that the presence of orchids was rarely limited by the availability of suitable OrM fungi at a landscape scale (McCormick and Jacquemyn, 2014). At the local scale, however, seed germination is often unpredictable, suggesting that OrM fungi are not regularly distributed within sites. Results from previous studies have, for example, shown that germination and seedling establishment in photosynthetic orchids often decline with increasing distance from adult orchids (Batty et al., 2001; Diez, 2007; Jacquemyn et al., 2007, 2012a,b; McCormick et al., 2012). Because most green adult orchids maintain associations with mycorrhizal fungi, this may create favorable microsites for seed germination near the rhizosphere of adult orchids (Cameron et al., 2008; Smith and Read, 2008; McCormick et al., 2016; Waud et al., 2016).

Mixed results have been obtained for fully mycoheterotrophic orchids, which are species that are non-photosynthetic throughout their entire life and solely depend on fungal carbon. In *Neottia nidus-avis*, for example, significantly higher seed germination and seedling establishment were observed in plots containing adult *N. nidus-avis* plants than in plots situated at least 5 m from the nearest adult, but there was no significant difference in seed germination between seed packets buried within 15 cm of the adults and packets buried at 15–25 cm from adult plants (McKendrick et al., 2002). In *Corallorhiza trifida*, on the other hand, there was no significant relationship between the extent of germination or seedling establishment and the presence of naturally distributed plants at a spatial scale of 1 m (McKendrick et al., 2000), suggesting that growth of *C. trifida* seedlings depends more on the ability of the fungal symbionts to transfer carbon from their ectomycorrhizal co-associates than on the distribution of conspecific adult plants.

In this study, we investigated the abundance and distribution of mycorrhizal fungi in bamboo litter and how they relate to spatial patterns of seed germination of the fully mycoheterotrophic orchid *Gastrodia confusoides*. A number of species of the genus *Gastrodia* exclusively inhabit bamboo forests and are parasitic on litter-decaying fungi to gain access to nutrients through the degradation of bamboo leaf and twig litter, while the litter-decaying fungi are most likely not dependent on this association for survival (Kinoshita et al., 2016). Given that the ground surface of bamboo forests is densely covered by bamboo leaf and twig litter, and litter-decaying fungi often show highly scattered patterns within the bamboo forest (Y-I Lee, pers. observation), we hypothesized that germination is likely more dependent on fungal abundance in the litter than on the distance to the nearest adult plant. To test this hypothesis, we used high-throughput DNA sequencing to compare the fungal communities associating with protocorms and adults to identify the mycorrhizal fungi promoting seed germination in *G. confusoides*. We further combined seed germination experiments with quantitative PCR (qPCR) analyses to investigate spatial variation in fungal abundance within the surrounding bamboo litter at different distances from adult plants and to assess how spatial variation in fungal abundance affected seed germination.

## Materials and Methods

### Study Species

The genus *Gastrodia* consists of about 66 species that are widely distributed across the Paleotropics, Oceania, and warm-temperate regions in Eastern Asia (Pridgeon et al., 2005; WCSP, 2014). All of them are all fully mycoheterotrophic plants that occur in moist and sheltered forests. *Gastrodia* species have been reported to associate with litter-decaying fungi and/or wood-decaying fungi (Kusano, 1911; Martos et al., 2009; Ogura-Tsujita et al., 2009; Dearnaley and Bougoure, 2010; Lee et al., 2015). Interestingly, several *Gastrodia* species, such as *Gastrodia appendiculata*, *Gastrodia confusoides*, and *Gastrodia nantoensis* occur predominantly in bamboo forests in Taiwan (Hsu, 2008), and the litter-decaying fungus, *Mycena* has been identified as their major mycorrhizal partner (Ogura-Tsujita et al., 2009; Lee et al., 2015).

**FIGURE 1:**
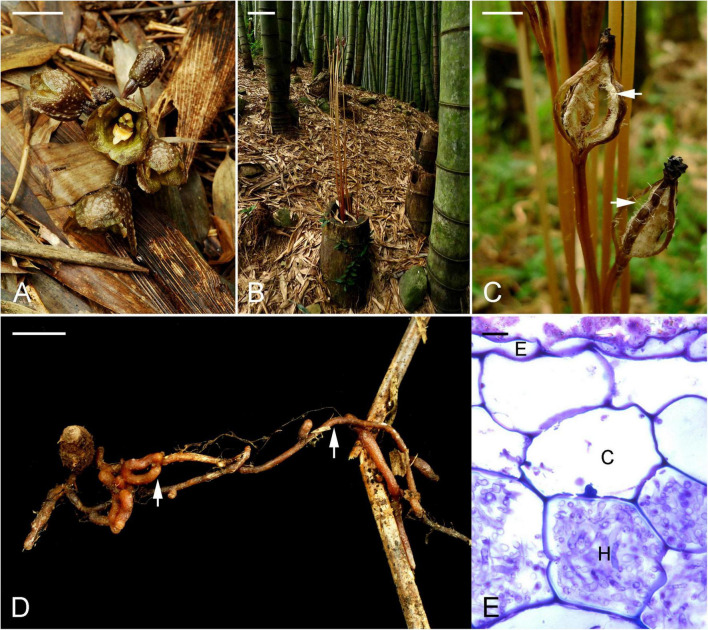
*Gastrodia confusoides* in its natural habitat. **(A)** Adult plants with blooming flowers growing between bamboo litter. Scale bar = 1 cm. **(B)** Stem elongation after flowers have been successfully pollinated. Scale bar = 10 cm. **(C)** After 2 weeks of pollination, mature fruits have opened, and released seeds (arrows). Scale bar = 1 cm. **(D)** The tube with mycorrhizal roots (arrows) in decaying bamboo litter. Scale bar = 1 cm. **(E)** Light microscopy section showing the presence of mycorrhizal fungi in cortical cells of roots. E, epidermal cells; C, cortical cells; H, fungal hyphae. Scale bar = 10 μm.

*Gastrodia confusoides* is a fully mycoheterotrophic perennial orchid that occurs mainly in bamboo forests in central Taiwan. The species generally flowers in September, producing short flowering stalks that vary in height between 2 and 5 cm (Figure 1A). After successful pollination, the flowering stalks elongate rapidly and reach a height between 20 and 50 cm (Figure 1B). Capsules ripen quickly 1 month after pollination, followed by dehiscence and seed dispersal in October (Figure 1C). Only flowers or elongated stalks with capsules of this fully mycoheterotrophic orchid can be found above-ground from September to October. After the seeds have been dispersed, successful development of underground tubers and roots depends on fungal associations with litter-decaying fungi (Figures 1D,E).

### Seed Germination Experiment

To investigate whether seed germination varied along the distance to the adult plant, a seed germination experiment was established in four plots using the seed packet method described by Rasmussen and Whigham (1993). Experiments were conducted in a bamboo forest located in Heping District, Taichung City, Taiwan at 1,000 m above sea level (24 23′92″N, 120 90′71″E). The bamboo forest was dominated by mature mōsō bamboo (*Phyllostachys edulis*) with only few understory plants, including *Polygonum japonicum* and *Deparia petersenii*. The bamboo forest has been planted and tilled by local farmers for harvesting bamboo shoots for over 50 years. The soils can be classified as Inceptisols, with mostly sandy loam with sand being the dominant particle. Soil pH of the four study plots ranged from 4.0 to 4.5.

**FIGURE 2:**
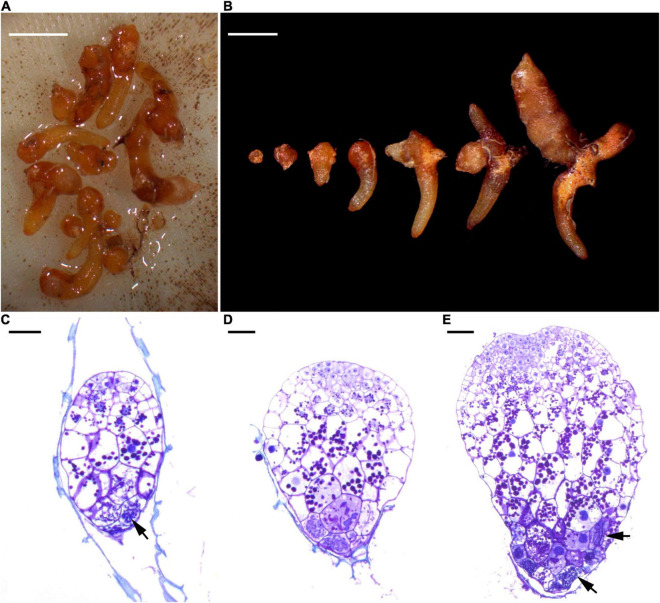
Different stages in the germination process of *Gastrodia confusoides* seeds and indications of the presence of mycorrhizal fungi. **(A)** Developing protocorms from a single packet at 12 months after sowing. Scale bar = 2 mm. **(B)** A selection of different stages of protocorms and young root tubers from the packet. Scale bar = 2 mm. **(C)** At the early stage of germination, fungal hyphae (arrow) have colonized the basal part of embryo. Scale bar = 50 μm **(D)** The enlarged embryo has ruptured the seed coat. Scale bar = 50 μm **(E)** The developing protocorm has elongated with the meristematic cells at the up region and the fungal colonization at the basal region. Fungal hyphae (arrows). Scale bar = 50 μm.

When seeds were mature at the beginning of October 2015, capsules were harvested from the studied populations. In order to exclude potential population effects that may create differences in seed quality, all seeds were pooled after harvesting and directly put into seed packets. Per seed packet, approximately 500 seeds were placed within a square of 53-μm mesh phytoplankton netting. Seed packets were buried into the litter layer at a depth of 5 cm in each cardinal direction (north, south, east, west) at five different distances (5, 50, 100, 200, 500 cm) around each sampled orchid adult (McCormick et al., 2016) in the same 10-m^2^ plot where roots and litter samples were taken (Supplementary Figure 1). Four adult orchids were selected without visible neighboring adult orchids within a radius of 5 m. In total, 5 (distances) × 4 (directions) × 4 (plots) = 80 seed packets were left in the ground for 1 year. In October 2016, seed packets were retrieved, gently washed and maintained moist in paper towel for 1 day until examination. In the laboratory, seed packets were gently rinsed with tap water, then opened and carefully checked under a stereo microscope to count the number of germinated seeds. As orchid seed germination stages can be variable, germination was considered to have occurred when clear signs of mycorrhiza formation were observed and rupture of testa by enlarging embryos was present (Figure 2).

### Sampling of Mycorrhizal Communities

Litter samples were collected in each cardinal direction (north, south, east, west) at five different distances (5, 50, 100, 200, 500 cm) around each sampled orchid adult (Supplementary Figure 1). At each sampling point, litter samples (ca. 10 g fresh weight of decaying bamboo leaves) were taken at a depth of 5 cm using a sterile sampling bag, and the litter samples were immediately stored at −80°C for subsequent analysis. Genomic DNA from litter samples was extracted using the PowerSoil^®^ DNA Isolation Kit (MO BIO Laboratories Inc., Carlsbad, CA). Additionally, at least three roots from each sampled orchid adult plant were surface sterilized with a 1% sodium hypochlorite solution for 30 s, followed by three 30-s rinse steps in sterile distilled water and microscopically checked for mycorrhizal colonization. Subsequently, the distal 5-cm portions of colonized roots were sectioned into 3–5 mm fragments, which were then pooled to create a homogeneous mycorrhizal root sample for each individual plant. The protocorms from seed packets in each plot were pooled because of the small amount of tissue. The protocorms were rinsed with water to remove excess soil and litter fragments and were surface sterilized as root samples described above. In each protocorm sample, 20 protocorms were pooled for DNA extraction. Afterward, DNA was extracted from 0.1 g mycorrhizal root fragments or protocorms using the Plant Genomic DNA Purification Kit as described by the manufacturer (GMbiolab Co., Ltd., Taichung, Taiwan). Altogether, there were four plots containing 88 unique samples (4 adult root samples, 4 protocorm samples, 80 litter samples) for DNA extraction and subsequent molecular analysis.

### Molecular Methods

The ITS1 region of the nuclear ribosomal RNA genes was amplified using the primer pair ITS1F and ITS2R (Supplementary Table 1; Adams et al., 2013). ITS1F/ITS2R has high amplification efficiency of a broad range of basidiomycetes and ascomycetes, and it produces a wide variety of OTUs corresponding to different orchid-associating mycorrhizal families (Waud et al., 2014). PCR was carried out in 20 μL reactions containing 10 ng of genomic DNA, 0.8 μL of each primer (5 μM), 2 μL of 2.5 mM dNTPs, 4 μL of 5x TransStart^®^ FastPfuBuffer (TransGen Biotech Co., Ltd., Beijing, China), and 0.4 μL of TransStart^®^ FastPfu Polymerase. The parameters of reactions consisted of an initial denaturation at 95°C for 3 min, then 35 cycles of 95°C for 30 s, and 55°C for 30 s and a final extension of 72°C for 45 s. The purified PCR products of the fungal internal transcribed spacer (ITS) plus the 5′ part of the 28S rDNA of *Mycena* or *Gymnopus* from our previous study on *Gastrodia* species (Lee et al., 2015) were included as positive control reactions, while negative control reactions (no template DNA) were included to verify target-specific amplification and the absence of contaminants. PCR products were separated by gel electrophoresis and amplicons within the appropriate size range were cut and purified using the AxyPrep DNA Gel Extraction Kit (Axygen Biosciences, Union City, CA) and quantified using QuantiFluor™ Fluorometer (Promega Corporation, Madison, WI). Samples were then pooled in equimolar concentrations and paired-end sequenced (2 × 250 bp) on an Illumina Miseq.

### Bioinformatics

Raw fastq files were demultiplexed and quality-filtered using QIIME (version 1.17) with the following criteria. First, 300 bp reads were truncated at any site receiving an average quality score < 20 over a 50 bp sliding window, discarding the truncated reads that were shorter than 50 bp. Second, reads with one or two nucleotide mismatches in primer matching measurements and reads containing ambiguous characters were removed. Third, only sequences that had an overlap longer than 10 bp were assembled according to their overlap sequence. Reads that could not be assembled were discarded.

Sequences obtained from the Illumina Miseq run were clustered into operational taxonomic units (OTUs) using the UPARSE algorithm implemented in USEARCH version 7 (Edgar, 2013). OTUs were clustered with 97% similarity cutoff using UPARSE (version 7.1)^1^ and chimeras were identified and removed using the UNITE UCHIME reference data set. The taxonomy of each ITS1 region was analyzed by RDP Classifier^2^ against the UNITE fungal ITS database using confidence threshold of 70%. Remaining OTUs were assigned taxonomic identities based on the top 10 BLAST (megablast algorithm) (Altschul et al., 1990) results of the OTU representative sequences (selected by UPARSE) using the GenBank nucleotide (nt) database (Benson et al., 2009), including uncultured/environmental entries. OTUs identified by BLAST were then manually screened for potential orchid-associating mycorrhizal families following a step-wise process. OTUs represented by short sequences (<150 bp) or having a low sequence similarity (<90%) with fungal species across their sequence length were removed. The results were put in an OTU table (sample × OTU table with each cell containing read numbers), which was used in the further analyses.

### Assessment of Fungal Abundance

Illumina Miseq sequencing revealed that the most abundant (in terms of read counts) OrM fungi associating with *Gastrodia confusoides* corresponded to two distinct saprotrophic non-rhizoctonia fungal taxa, i.e., *Mycena* (OTU3) and *Gymnopus* (OTU5). For both OTUs, a real-time quantitative PCR (qPCR) assay was developed and validated based on the internal transcribed spacer (ITS) 2 region (Supplementary Table 1). The qPCR assays were utilized to quantify *Mycena* (OTU3) and *Gymnopus* (OTU5) DNA from each protocorm, root or litter DNA sample on an ABI QuantStudio 6 Flex real-time PCR instrument (Life Technologies, Carlsbad, CA, United States). Reactions were performed in a total volume of 25 μL consisting of 12.5 μL of iQ SYBR Green PCR Super Mix (Bio-Rad Laboratories, Hercules, CA, United States), 1.0 μL DNA template, 9.0 μL H_2_O and 1.25 μL (10 mM) of each forward and reverse primer, depending on the target OTU (Supplementary Table 1). Amplifications were run as follows: initial denaturation for 5 min at 95°C, followed by 40 cycles of 15 s of denaturation at 94°C, 30 s of annealing at 59°C (OTU3 and OTU5), and 30 s of elongation at 72°C. Each sample extract was amplified in duplicate and quantified using a standard curve amplified in triplicate. Standard curves (range, 1.0E + 02 to 1.0E + 06 molecules μ1^–1^) were generated using five 10-fold dilutions of target DNA amplified from protocorms (OTU3) or roots (OTU5). A melting curve analysis was performed after each analysis to confirm product specificity (Supplementary Figures 2A,C). In addition, amplification accuracy was verified by Sanger sequencing of a number of generated amplicons. In this way, product identity was confirmed for all samples considered to be positive using the qPCR assays (Ct value < 29 and a single melting peak at the expected melting temperature; Supplementary Figure 2). Additional positive (DNA extract from protocorms colonized with OTU3 or roots colonized with OTU5) and negative control reactions (no template DNA) were included in triplicate with all analyses to verify target-specific amplification and the absence of contaminants, respectively. The positive control showed that the amplification of target DNA was accurate and sensitive, while the negative control showed that no contaminants were present.

### Data Analysis

To investigate whether fungal richness (the number of OTUs) differed between protocorms, adults, and soil samples collected at different distances from adult plants, we used a Linear Mixed Model, with the number of OTUs as dependent variable and sample type as fixed factor. Plot was included as a random factor in the analyses. To see how fungal communities differed between protocorms, adult plants, and soil samples, non-metric multidimensional scaling (NMDS) was performed using read abundance data as explanatory variables and the Bray–Curtis coefficient as distance measure. Additionally, permutational multivariate analysis of variance (PERMANOVA; Anderson, 2001) was performed to test whether the fungal communities differed significantly between protocorm, adult, and soil samples. All analyses were performed in the vegan package (Oksanen et al., 2013) in R (R Core Team, 2020). The same analyses were used to test whether fungal communities found in the soil differed between the four sampling plots and between cardinal directions and distances within plots.

To investigate spatial variation in mycorrhizal abundance and seed germination, we used Generalized Linear Mixed Models and Linear Mixed Models. First, a Linear Mixed Model was used to test the hypothesis that fungal abundance was related to the distance of the nearest adult plant. Fungal concentration (expressed in terms of molecules per μL) was used as dependent variable, while distance to adult plants was used a fixed factor. Plot was included in the model as a random factor. Analyses were performed separately for the main fungus associating with protocorms and the fungus associating with adult plants. A Generalized Linear Mixed Model was used to see whether seed germination (at least one seed developing into protocorm) was related to the distance of the nearest adult plant or to the abundance of key fungi (*Mycena* and *Gymnopus*) in the litter. Presence/absence of at least one protocorm in seed packets was used as dependent variable, while plot was included as a random effect in the model. In a second analysis, we related germination success (i.e., the percentage of seeds developing into a protocorm) to the distance of the nearest adult plant and the abundance of fungi in the soil.

## Results

### Mycorrhizal Associations

The quality-filtered Miseq data set contained samples from protocorms, adult roots, and surrounding bamboo litter. The numbers of OTUs were close to saturation after 15,000 sequence counts (Supplementary Figure 3). The four composite protocorm samples comprised a total of 129 OTUs (172,552 sequences), while the 4 adult root samples comprised 427 OTUs (168,843 sequences) (Supplementary Table 2). The surrounding 80 bamboo litter samples comprised 3,925,341 sequences with a total of 1,640 OTUs (Supplementary Table 4, excluding OTUs with < 1,000 total sequences). The number of OTUs per sample differed significantly (χ^2^ = 100.9, *p* < 0.0001) between protocorms, adults, and soil samples. While soil samples contained on average > 210 OTUs, the number of OTUs was much lower in protocorms (20.8 ± 4.5) and adult plants (47.0 ± 24.01). Soil samples taken at a distance of 200 and 500 cm contained significantly (*p* < 0.01) more OTUs than samples taken at 5 and 100 cm (Supplementary Figure 4).

**FIGURE 3:**
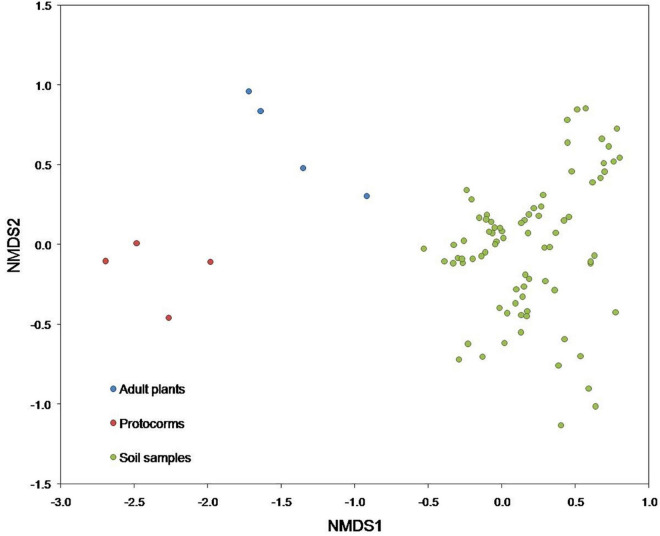
Non-metric multidimensional scaling (NMDS) plot illustrating differences in fungal communities detected in protocorms, adult plants and soil samples. Each data point represents an individual protocorm (red), adult (blue), or soil (green) sample.

At the class level, the predominant fungi belonged to Agaricomycetes, Sordariomycetes, Leotiomycetes, Eurotiomycetes, Archaeorhizomycetes, accounting for 31.7, 25.9, 11.97, 8.34, and 8.22%, respectively (Supplementary Figure 5A). At the order level, the five most abundant orders were Agaricales, Trechisporales, Helotiales, Hypocreales, and Chaetothyriales, accounting for 15.93, 15.8, 15.04, 11.02, and 10.6%, respectively (Supplementary Figure 5B). At the family level, the predominant fungi belonged to Hydnodontaceae, Mortierellaceae, Myxotrichaceae, Herpotrichiellaceae, and Archaeorhizomycetaceae, accounting for 18.94, 12.38, 12, 11.86, and 4.72%, respectively (Supplementary Figure 5C). Fungal communities differed significantly [*F*_(2_, _85)_ = 7.30, *p* < 0.001] between protocorms, adult plants, and soil samples (Figure 3). Besides, fungal communities in the soil also differed significantly [*F*_(3_, _76)_ = 6.95, *p* < 0.0001] between the four plots that were sampled (Supplementary Figure 6), but no spatial differences in fungal communities were found within plots.

Most orchid mycorrhizal sequences were related to members of *Mycena* (one OTU—164,772 sequences—95.5%) in the protocorm samples, and *Gymnopus* (one OTU -90,028 sequences—53.3%) in the adult root samples. It is noteworthy that fungal partners differed between protocorm and adult stages in *G. confusoides*, in which a single *Mycena* (OTU3) was dominant and occurred exclusively in protocorms, and one prevailing *Gymnopus* (OTU5) that occurred solely in roots (Supplementary Table 2 and Supplementary Figure 7). Raw sequences reads were deposited in NCBI Sequence Read Archive database (accession numbers PRJNA753675 and PRJNA778455).

qPCR standard curves based on known concentrations of target DNA showed a linear correlation (OTU3, *r*^2^ = 0.998; OTU5, *r*^2^ = 0.997) between log values of input DNA and qPCR threshold cycles over at least five orders of magnitude (Supplementary Figures 2B,D), enabling accurate quantification of our target fungi in terms of target DNA molecules (mol) per μL DNA extract (Supplementary Table 3). The highest concentrations of target OrM DNA were obtained from *G. confusoides* protocorm and root samples, with the average OTU3 concentration being 640,129.5 mol μ1^–1^ DNA extract (SE = 43787.6 mol μ1^–1^) and the average OTU5 concentration being 484626.5 mol μ1^–1^ DNA extract (SE = 62,923 mol μ1^–1^), respectively. For these two target OTUs, no significant relationship between the distance from the orchid plant and fungal concentration was observed in bamboo litter surrounding the orchid plant (χ^2^ = 4.40 and 6.39, *p* > 0.05 for OTU3 and OTU5, respectively) (Supplementary Figure 8). In both cases, plot explained a minor part (<10%) of the total variation, indicating that the results were consistent among plots.

### Germination

**FIGURE 4:**
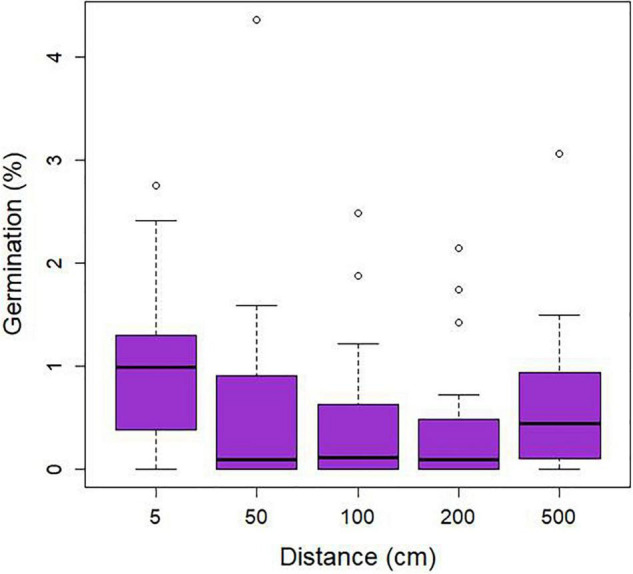
Relationship between the percentage seed germination of *Gastrodia confusoides* and distance from adult plants (cm) measured in four plots in bamboo forest in Taiwan.

**FIGURE 5:**
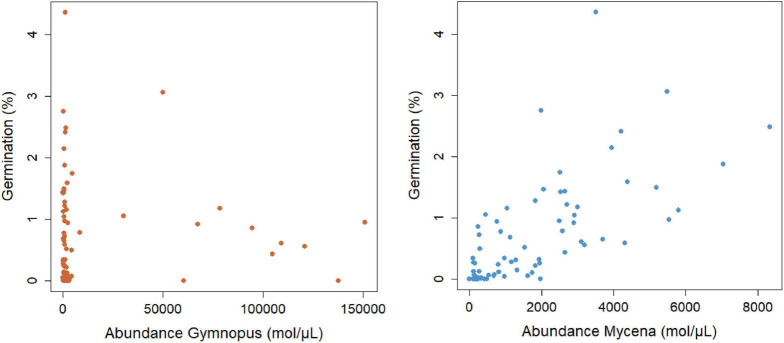
Relationship between the abundance of *Mycena* (OTU3) and *Gymnopus* (OTU5) DNA molecules μL^–1^ DNA extract and percentage seed germination of *Gastrodia confusoides*. Seed germination was expressed as the percentage of seeds developing into a protocorm.

Seed germination was regularly encountered at the study site and distinct stages of seedling development (protocorms and young root tubers) were observed (Figures 2A,B). The percentage of packets containing germinating seeds varied between 70% in plot 1, 90% in plot 2, 85% in plot 3, and 65% in plot 4. On average, 3.2 protocorms (0.64% seed germination) were found within individual seed packets. Analysis of data for germination of seed packets showed no significant effect (χ^2^ = 4.58; *p* = 0.33) of distance to adult plants within the 10-m^2^ plots (Figure 4), nor was there any relationship between the four directions in which seed packets were buried in the litter (data not shown). A strong, significant relationship (χ^2^ = 51.91; *p* < 0.001) was observed between seed germination and abundance of *Mycena* in the litter, but not between seed germination and abundance of *Gymnopus* (χ^2^ = 0.04, *p* = 0.84) (Figure 5).

## Discussion

### Mycorrhizal Communities Associating With *Gastrodia confusoides*

Autotrophic and mycoheterotrophic orchids tend to associate with different sets of mycorrhizal fungi (Jacquemyn and Merckx, 2019; Wang et al., 2021). Whereas most autotrophic orchids typically associate with a wide variety of fungi including so-called rhizoctonia fungi (i.e., members of Tulasnellaceae, Ceratobasidiaceae, and Serendipitaceae), partially and fully mycoheterotrophic orchids associate predominantly with ectomycorrhizal fungi (Tesitelova et al., 2015; Yagame et al., 2016; Ogura-Tsujita et al., 2021) and saprotrophic fungi (Lee et al., 2015; Ogura-Tsujita et al., 2018). Our results are in line with these observations and showed that the dominant fungi associating with *G. confusoides* were saprotrophic fungi of the Mycenaceae and Marasmiaceae (Supplementary Table 2).

These results further confirm previous reports that have shown that fungi from Mycenaceae and Marasmiaceae are the two major fungal families associating with *Gastrodia* species (Ogura-Tsujita et al., 2009; Dearnaley and Bougoure, 2010; Lee et al., 2015; Kinoshita et al., 2016). Individuals of *Gastrodia nipponica* inhabiting broad leaved forests were shown to simultaneously associate with ectomycorrhizal fungi from Russulaceae and Sebacinaceae, suggesting that orchid mycorrhizal communities in *Gastrodia* species can vary depending on the vegetation type where the species grow. A similar result was found in *Gastrodia elata*, which is mainly found in broadleaved forests. Chen et al. (2019) showed that besides Mycenaceae, a broader spectrum of fungal associations, such as *Resinicium*, *Hyphodontia*, *Sistotrema*, *Tricholoma*, and *Russula* was detected in the juvenile stage of *G.elata*. A higher fungal diversity in the juvenile stage may enable *G. elata* to access a range of carbon pools for germination and/or protocorm development from various mycobiont communities in different vegetation types.

The *Mycena* (OTU3) sequences isolated from *G. confusoides* protocorms shared high sequence similarity with sequences isolated from *G. nantoensis* and *G. appendiculata* growing in other bamboo forests (Lee et al., 2015). These results suggest that this *Mycena* strain is particularly adapted to grow in bamboo forest, where it decomposes bamboo litter, and therefore could be shared among *Gastrodia* species. It has been reported that only a limited group of saprotrophic fungi can occur in bamboo forests in Japan, and the fungal community in bamboo forests is different from that in other forest types (Shidei, 1974; Yashima and Nose, 2010). These results suggest the adaptation of “the bamboo forest *Gastrodia* species” to particular fungal groups specific to bamboo thickets.

As compared to the protocorm (OTU3, 95.5%), the adult root of *G. confusoides* possessed a higher diversity of fungi (OTU5, 53.3% of all sequences with the remaining 46.7% of various fungal communities) (Supplementary Table 2). Most of the remaining fungal OTUs belonged to Ascomycota that were not typical OrM fungi, except for *Fusarium oxysporum*. Although a *F. oxysporum* strain has been confirmed to serve as an OrM fungus in a terrestrial orchid, *Bletilla striata* (Jiang et al., 2019), the role of *F. oxysporum* in *Gastrodia* mycorrhizal symbiosis is not clear.

### Shift in Fungal Partners Between Different Developmental Stages

Although it is clear that mycorrhizal interactions play a crucial role in the life cycle of orchids (Rasmussen, 1995), there is remarkable variation in the number of fungi orchids associate with, ranging from highly specific interactions with one or a few OrM fungi (McCormick et al., 2009) to very broad interactions with orchids associating with multiple fungi simultaneously (e.g., Jacquemyn et al., 2012c; De Long et al., 2013; Waud et al., 2017; Shefferson et al., 2019). Furthermore, there is growing evidence that orchid mycorrhizal interactions are not stable, but can vary substantially both in space (Lin et al., 2020; Xing et al., 2020) and time (Bidartondo and Read, 2008; Ventre-Lespiaucq et al., 2021), with some species continuously associating with the same partner while others show almost complete switching of partners through space or time (Ventre-Lespiaucq et al., 2021). Spatial and temporal turnover may reflect variation in ecological conditions or in fungal availability. Temporal turnover can occur throughout the growing season, between subsequent years (e.g., McCormick et al., 2006; Ercole et al., 2015; Ojá et al., 2015), or between ontogenetic stages (e.g., McCormick et al., 2004; Bidartondo and Read, 2008; Jacquemyn et al., 2011). For example, *Tipularia discolor* adults associate with a variety of fungi, while fungal diversity detected in protocorms is much lower (McCormick et al., 2004). A recent meta-analysis has shown that temporal turnover of mycorrhizal partners occurs frequently in orchids and that in most cases partial replacement, whereby an individual retains a subset of its fungal partners and replaces others, was the most frequent scenario (Ventre-Lespiaucq et al., 2021).

Our high-throughput sequencing data demonstrated that *G. confusoides* switched fungal partners from the protocorm stage (Mycenaceae) to the adult stage (Marasmiaceae). A similar shift in fungal partner across ontogenetic stages has been observed in *G. elata* using an *in vitro* culture system, in which *Mycena* promoted seed germination and the further development of the tuber required the association with *Armillaria* (Xu and Guo, 2000). However, high-throughput sequencing of both juvenile and adult stages of *G. elata* showed that the juvenile stage associated with more diverse fungal groups than *Mycena* alone (Chen et al., 2019). A similar pattern of total replacement has been found in *Cyrtosia septentrionalis*, another mycoheterotrophic orchid associated with wood-decaying fungi. In this case, *Physisporinus* induces seed germination, while the adult stage associates with *Armillaria* (Umata et al., 2013).

Complete partner turnover seems to occur primarily in subtropical Asian mycoheterotrophic orchids associated with litter- or wood-decaying fungi, but not in temperate mycoheterotrophic orchids. In temperate forests, mycoheterotrophic orchids, such as *Cephalanthera austiniae*, *Corallorhiza* species, and *Neottia nidus-avis* usually associate with narrow clades of ectomycorrhizal fungi during their entire life cycle (Taylor and Bruns, 1997; McKendrick et al., 2000, 2002). Although the precise reasons for mycorrhizal switching are not well understood, previous research has shown that shifts in fungal partners are risky and may come with a cost, constraining the population dynamics of orchids (McCormick et al., 2006; Ogura-Tsujita et al., 2021; Ventre-Lespiaucq et al., 2021). In the case of mycoheterotrophic orchids, switching partners may occur when it provides benefits in terms of resource acquisition (Ogura-Tsujita et al., 2021). For example, in the fully mycoheterotrophic *G. elata*, changing fungal partners from *Mycena* to *Armillaria* may enable the utilization of a larger carbon pool, i.e., the wood in the forest (Ogura-Tsujita et al., 2021). Similar differences in resource acquisition abilities may explain the observed switch in mycorrhizal partners in protocorms and adult plants of *G. confusoides*.

### Abundance and Spatial Distribution of Mycorrhizal Fungi and Seed Germination

The distribution and abundance of suitable OrM fungi in the soil are critical factors determining seed germination and hence the spatial distribution of orchids within sites (McCormick and Jacquemyn, 2014). Whereas a number of studies have shown strong relationships between the presence/absence of adult plants and seed germination (McKendrick et al., 2000, 2002; Batty et al., 2001; Diez, 2007; Jacquemyn et al., 2007, 2012a,b), we found no significant relationship between the distance from the mother plant and germination (Figure 4), indicating that locations closer to adult plants are not necessarily locations supporting higher abundances of mycorrhizal fungi required for seed germination. This can be explained by the fact that adults plants almost exclusively associate with *Gymnopus*, which was completely absent in germinating seeds and protocorms. We also found no correlation between seed germination and the abundance of *Gymnopus* (OTU5) (Figure 5), suggesting that this fungus is not involved in seed germination, but only starts to play a role in the subsequent growth of seedlings into adult plants. In contrast, protocorms associated mainly with *Mycena* (OTU3) and in this case there was a highly significant, positive relationship between seed germination and the abundance of *Mycena* (Figure 5), indicating that the local distribution of this fungus is the determining factor driving spatial patterns of seed germination.

These differences in seed germination patterns between autotrophic and mycoheterotrophic orchids may be explained by the nutritional needs of different groups of OrM fungi they associate with. In orchids associating with rhizoctonia fungi, the accumulation of fungi around adults plants and the associated strong relationship between germination and the location of adult plants (Waud et al., 2016) may be explained by the fact that the orchids contribute carbon to and hence to some extent support the fungi, although clear evidence showing that orchids contribute carbon to the fungi is still very limited (Cameron et al., 2008; Liebel et al., 2015). In orchids associating with ectomycorrhizal fungi, the fungi are supported by trees, and hence the distribution and abundance of the orchids is for a large part related to the distribution and abundance of the fungi and their ectomycorrhizal trees (McCormick et al., 2009). In bamboo forests, the ground is usually covered with a thick layer of bamboo litter. Since *Mycena* and *Gymnopus* are efficient decomposers of bamboo leaves, sticks and trunks, they are expected to be widely distributed within the bamboo forest.

## Conclusion

Seed germination and subsequent growth to an adult plant in the mycoheterotrophic orchid *G. confusoides* represents a two-stage process that is accompanied by a switch in mycorrhizal partners. Our results further show that the main mycorrhizal fungi associating with protocorms and adult plants are randomly distributed within the bamboo forest and independently from *G. confusoides* adults. Future research should investigate in more detail the functional and physiological basis of the interaction with both fungi and assess the cost of switching. In particular, more details about resource transport patterns between symbionts are needed to understand why adult plants switch to a different partner.

## Data Availability Statement

The datasets presented in this study can be found in online repositories. The names of the repository/repositories and accession number(s) can be found below: SRA, PRJNA753675, PRJNA778455.

## Author Contributions

Y-IL designed the research. Y-YL and Y-HC performed the molecular analyses. Y-IL, Y-YL, and MB performed bioinformatic analyses. HJ conducted statistical analyses. Y-IL and HJ wrote the manuscript. All authors contributed to the article and approved the submitted version.

## Conflict of Interest

The authors declare that the research was conducted in the absence of any commercial or financial relationships that could be construed as a potential conflict of interest.

## Publisher’s Note

All claims expressed in this article are solely those of the authors and do not necessarily represent those of their affiliated organizations, or those of the publisher, the editors and the reviewers. Any product that may be evaluated in this article, or claim that may be made by its manufacturer, is not guaranteed or endorsed by the publisher.

## Footnotes

1
http://drive5.com/uparse/

2
http://rdp.cme.msu.edu/
