# Supplementary material for: Mycorrhizal Switching and the Role of Fungal Abundance in Seed Germination in a Fully Mycoheterotrophic Orchid, Gastrodia confusoides
Source: Front Plant Sci. 2022 Jan 13;12:775290. doi: 10.3389/fpls.2021.775290 (PMC8792533; doi:10.3389/fpls.2021.775290)

Supplementary Material Tables S1, S2, S3and Figs S1, S2, S3, S4, S5, S6, S7 & S8

Article title: **Mycorrhizal switching and the role of fungal abundance in seed germination in a fully mycoheterotrophic orchid, *Gastrodia confusoides***

Authors: Yuan-Yuan Li, Margaux Boeraeve, Yu-Hsiu Cho, Hans Jacquemyn and Yung-I Lee

Table S1 List of primers used in this study.

Primer name	Forward primer (5-3')	Reverse primer (5-3')
ITS1F/ITS2R	CTTGGTCATTTAGAGGAAGTAA	GCTGCGTTCTTCATCGATGC
OTU3	CCTGGTACTGATGCTGGCCCTTTAAC	GGGGAAGGACTTTTGCAAGCTCAAGC
OTU5	GTTGCTGCTGGCCTCTTACGAGG	CTCGCTAGGTCGAAACCTATAAAGCC

Table S2 Summary of fungal operational taxonomic units (OTUs)[a] and their frequencies[b] detected in orchid protocorms and roots using the Illumina Miseq platform[c].

OTU Identifier	Closest GenBank Match			BLAST Identity	Protocorm		Root	
	Accession	Length (bp)	Match (%)		Sequences	Percent (%)	Sequences	Percent (%)
**Otu3**	**KP238199**	**200**	**99**	**Uncultured mycorrhizal fungus *Mycena***	**164772**	**95.5**	**0**	**0**
Otu1194	KP238193	198	99	Uncultured mycorrhizal fungus *Mycena*	0	0	127	0.1
**Otu5**	**AY263425**	**184**	**100**	***Gymnopus melanopus***	**0**	**0**	**90028**	**53.3**
Otu141	GU258271	135	88	*Lactarius subgerardii*	4805	2.8	0	0
Otu731	LC013341	200	100	Uncultured *Marasmiellus*	302	0.2	0	0
Otu168	MK579177	200	100	*Lecanicillium fungicola*	177	0.1	0	0
Otu125	KT728346	196	97	Uncultured *Cladophialophora* clone	442	0.3	3565	2.1
Otu26	KT728346	200	100	Uncultured *Cladophialophora* clone	846	0.5	1872	1.1
Otu78	KT777711	198	99	*Cladophialophora* sp.	219	0.1	1206	0.7
Otu108	AB986343	198	99	*Cladophialophora floridana*	0	0	328	0.2
Otu33	KR093936	185	100	*Cladophialophora* sp.	0	0	1513	0.9
Otu920	AB986343	186	94	*Cladophialophora floridana*	0	0	301	0.2
Otu225	KY589655	165	100	*Cladophialophora* sp.	0	0	219	0.1
Otu252	LS989817	147	94	Uncultured Trechisporales	0	0	1125	0.7
Otu380	LS989817	147	94	Uncultured Trechisporales	0	0	1302	0.8
Otu692	LS976138	152	99	Uncultured Agaricomycetes	0	0	345	0.2
Otu101	MK794263	200	100	*Pochonia chlamydosporia*	0	0	3329	2.0
Otu758	LS988381	143	95	Uncultured Sordariales	0	0	260	0.2
Otu49	KT777727	199	99	*Exophiala* sp.	0	0	12832	7.6
Otu18	MK536752	200	100	*Mortieriella* sp.	0	0	332	0.2
Otu20	MK536908	200	100	*Mortierella* sp.	0	0	547	0.3

OTU Identifier	Closest GenBank Match			BLAST Identity	Protocorm		Root	
	Accession	Length (bp)	Match (%)		Sequences	Percent (%)	Sequences	Percent (%)
Otu109	KF225840	200	100	Uncultured *Mortierella* clone	0	0	492	0.3
Otu587	MT521775	200	100	*Mortierella* sp.	0	0	384	0.2
Otu90	MH864068	197	99	*Chloridium* sp.	0	0	157	0.1
Otu129	MF330141	184	99	*Chaetothyriales* sp.	0	0	612	0.4
Otu21	MT560381	200	100	*Fusarium oxysporum*	0	0	36311	21.5
Otu363	MH378889	200	100	*Gliocladiopsis* sp.	0	0	162	0.1
Otu39	KY102937	200	100	*Saitozyma podzolica*	0	0	174	0.1
Otu550	KY588536	165	100	*Gamsomyces longisporus* clone	0	0	489	0.3
Otu647	KP814196	128	91	*Ceraceomyces* cf. serpens	0	0	379	0.2
Otu473	MT020865	156	98	*Bactrodesmium longisporum*	0	0	1111	0.7
Otu1456	MT020867	158	99	*Bactrodesmium stilboideum*	0	0	177	0.1
Otu34	MK024241	200	100	*Trichoderma pubescens*	0	0	630	0.4
Otu206	MT530250	198	99	*Trichoderma sulphureum*	0	0	493	0.3
Otu859	MH864916	119	86	*Penicillium lagena*	0	0	163	0.1
Otu1205	MT594380	145	87	*Penicillium* sp.	0	0	142	0.1
Otu83	MK267766	200	100	*Gloeotinia* sp.	0	0	273	0.2
Otu130	JX317196	196	98	Uncultured Helotiales	0	0	1196	0.7
Otu840	MK404664	62	98	*Bresadolia* sp.	0	0	149	0.1
Otu1074	MN431307	56	97	Sordariomycetes sp.	0	0	139	0.1
Otu159	MG707450	174	92	Uncultured Auriculariales	0	0	301	0.2
Otu1126	LS985507	147	100	Uncultured Ophiostomataceae	0	0	154	0.1
Otu981	LS990715	153	99	Uncultured Mucoromycotina	0	0	196	0.1
Otu172	MF531168	122	100	Uncultured Basidiomycota	0	0	146	0.1
Otu773	MK247897	191	100	Xylariaceae sp.	0	0	224	0.1

OTU Identifier	Closest GenBank Match			BLAST Identity	Protocorm		Root	
	Accession	Length (bp)	Match (%)		Sequences	Percent (%)	Sequences	Percent (%)
Otu98	MT832997	194	100	Paecilomyces sp.	0	0	365	0.2
Otu2	MK770284	190	95	Uncultured Trechisporales	0	0	242	0.1
Otu6	MH430368	175	97	Sordariomycetes sp.	0	0	154	0.1
Otu1541	KY104258	178	88	*Microsporomyces bloemfonteinensis*	0	0	150	0.1
Otu229	MT609901	200	100	*Cladosporium cladosporioides*	0	0	169	0.1

OTUs that quantitative PCR (qPCR) primers were designed for are highlighted in bold. [a]OTUs were defined at 3% sequence dissimilarity using the UPARSE pipeline described in Edgar (2013). Only OTUs representing fungal taxa were retained during analysis and only those OTUs with >100 total sequences are included here. [b]OTU frequencies are indicated by total number of sequences obtained and the percentage of total sequences that each OTU was detected upon. [c]Orchid protocorms and roots were processed as described in the Materials and Methods section.

Table S3 Mean concentration of OTU3 (*Mycena*) and OTU5 (*Gymnopus*) DNA (mol µl^{-1}) including standard deviation of the mean quantified using qPCR analysis of *Gastrodia confusoides* protocorms, roots and surrounding bamboo litter samples obtained in four cardinal directions and at five radial distances (5, 50, 100, 200 and 500 cm).

Mycena (OTU3) DNA Concentration (mol µl^{-1})							
		**Plot 1**	**Plot 2**	**Plot 3**	**Plot 4**	**Mean**	**SE**
**Adult roots**		0	0	0	0	0	0
**Protocorms**		634671	522391	674093	729363	640129.5	43787.6
**East**	**5 cm**	109	1048	2588	2052	1449.25	549.1
	**50 cm**	144	1836	3514	0	1373.5	826.3
	**100 cm**	0	107	2698	0	701.25	666.1
	**200 cm**	157	330	694	820	500.25	154.5
	**500 cm**	1297	2892	1327	863	1594.75	445.2
**West**	**5 cm**	758	1171	329	288	636.5	207.4
	**50 cm**	243	2494	684	485	976.5	513.8
	**100 cm**	202	7043	8339	982	4141.5	2072.4
	**200 cm**	274	808	204	173	364.75	149.2
	**500 cm**	209	3188	5187	1950	2633.5	1047.7
**South**	**5 cm**	5797	4210	3002	2919	3982	673.1
	**50 cm**	4390	1978	3101	251	2430	877.6
	**100 cm**	1737	1134	124	274	817.25	378.8
	**200 cm**	0	2517	545	182	811	579.8
	**500 cm**	449	5483	313	973	1804.5	1234.3
**North**	**5 cm**	2661	1987	1941	2661	2312.5	201.4

Gymnopus (OTU5) DNA Concentration (mol µl^{-1})							
		**Plot 1**	**Plot 2**	**Plot 3**	**Plot 4**	**Mean**	**SE**
**Protocorms**		0	0	0	0	0	0
**Adult roots**		479004	599090	549485	310927	484626.5	62922.9
**East**	**5 cm**	2600	1821	8499	436	3339	1777.2
	**50 cm**	1188	950	1062	462	915.5	158.7
	**100 cm**	137840	1176	894	340	35062.5	34259.6
	**200 cm**	697	3043	4456	626	2205.5	937.1
	**500 cm**	217	67305	617	551	17172.5	16711.1
**West**	**5 cm**	2522	349	601	4300	1943	923.4
	**50 cm**	94620	151050	0	1158	61707	37125.2
	**100 cm**	3043	807	1494	1391	1683.75	477.6
	**200 cm**	921	432	330	198	470.25	157.6
	**500 cm**	787	120772	676	568	30700.75	30023.7
**South**	**5 cm**	208	1046	78395	436	20021.25	19458.7
	**50 cm**	2266	362	109096	3170	28723.5	26797.2
	**100 cm**	1349	323	1532	2026	1307.5	357.9
	**200 cm**	1231	4548	3013	210	2250.5	960.1
	**500 cm**	30296	50116	252	137	20200.25	12238.4
**North**	**5 cm**	0	257	371	104747	26343.75	26134.5

**50 cm**	124	116	158	198	149	18.6	**50 cm**	224	476	2600	1219	1129.75	533.6
**100 cm**	134	424	1526	4309	1598.25	952.1	**100 cm**	2433	2037	1946	994	1852.5	305.1
**200 cm**	2528	1836	3946	248	2139.5	768.3	**200 cm**	306	1859	669	540	843.5	346.7
**500 cm**	140	3698	5535	1628	2750.25	1180.6	**500 cm**	60489	540	828	251	15527	14987.7

Fig. S1 Radial sampling plan applied for the seed germination experiment and the collection of bamboo litter surrounding *Gastrodia confusoides* individuals, where each point indicates the position of sampling mapped according to their distance (cm) and direction (N, S, E, W) from a central adult orchid plant.

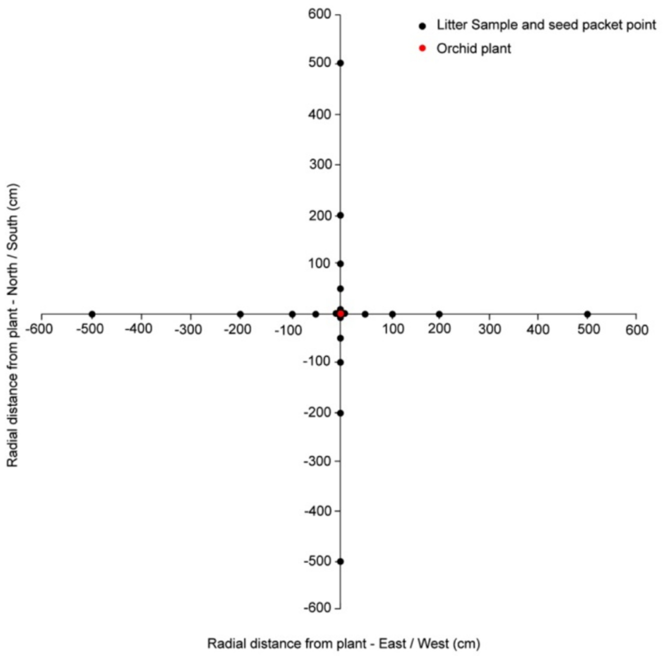

Fig. S2 Quantitative PCR results for OTU3 (*Mycena*) and OTU5 (*Gymnopus*)detected in *Gastrodia confusoides* protocorms or roots, including melting curve analyses where the negative derivative of fluorescence with respect to temperature is plotted as derivative reporter (-R') vs temperature to obtain a graphical representation of the melting peaks (A, C) and the standard curves used for the quantification of target DNA in biological complex samples using qPCR results obtained from amplification of a 10-fold dilution series of target DNA in triplicate (B, D).

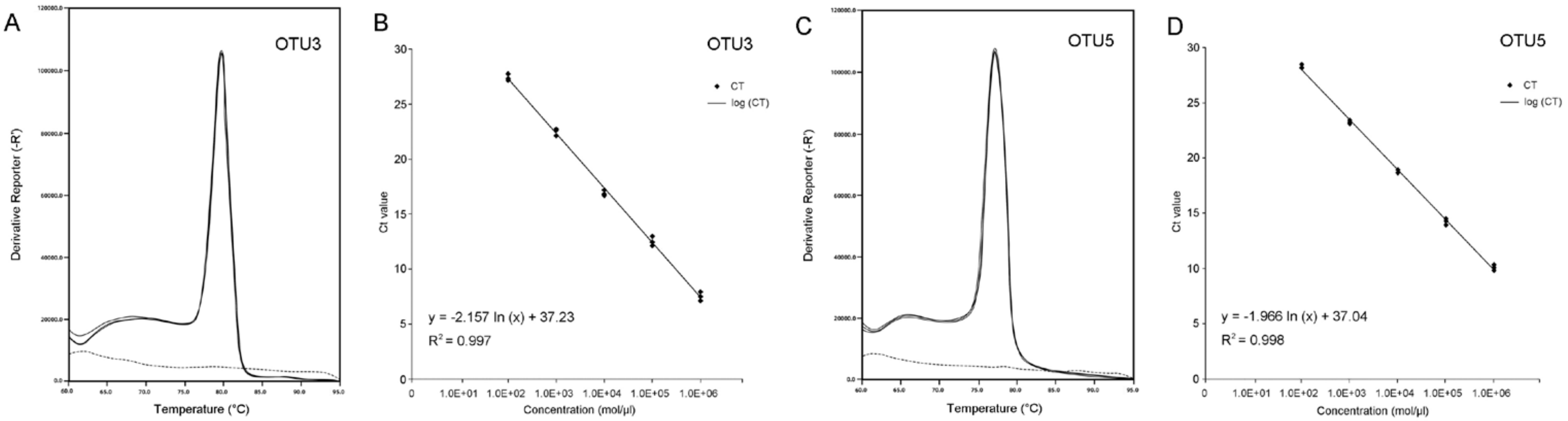

Fig. S3 OTU rarefaction curves by randomly selecting smaller fractions of reads 100 times.

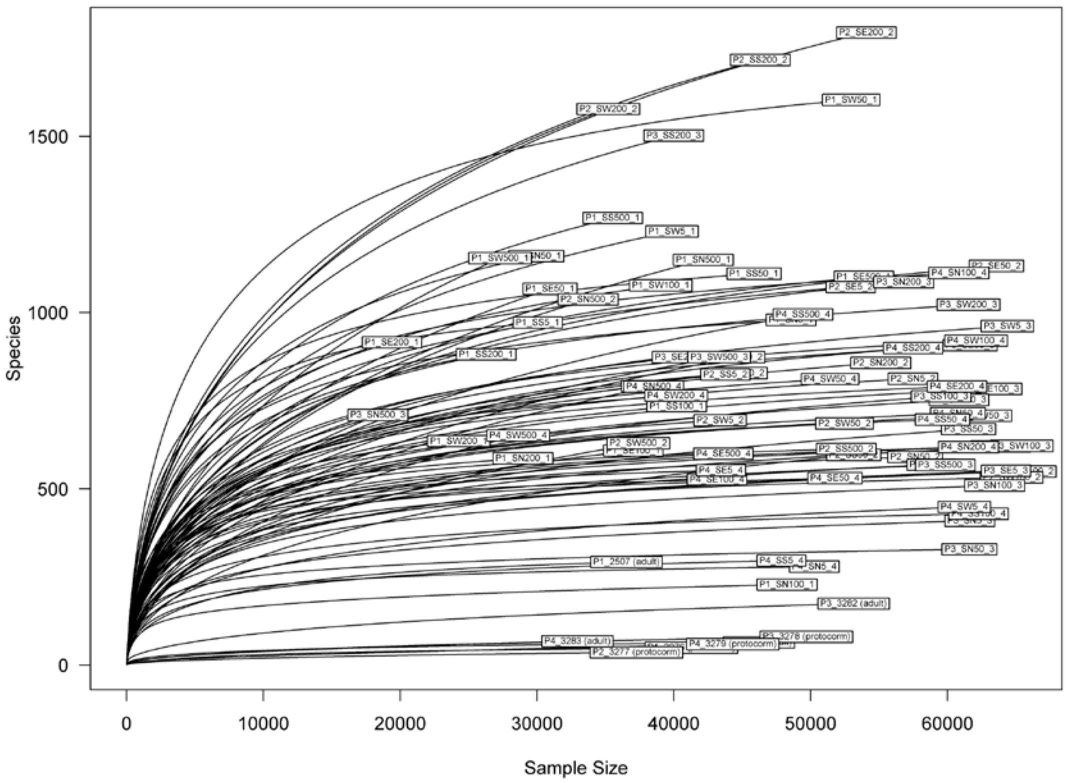

Fig. S4 Differences in OTU richness between protocorm, adults and soil samples taken at different distances from adult plants (D5: 5cm, D50: 50cm, D100: 100 cm, D200: 200 cm, D500: 500 cm).

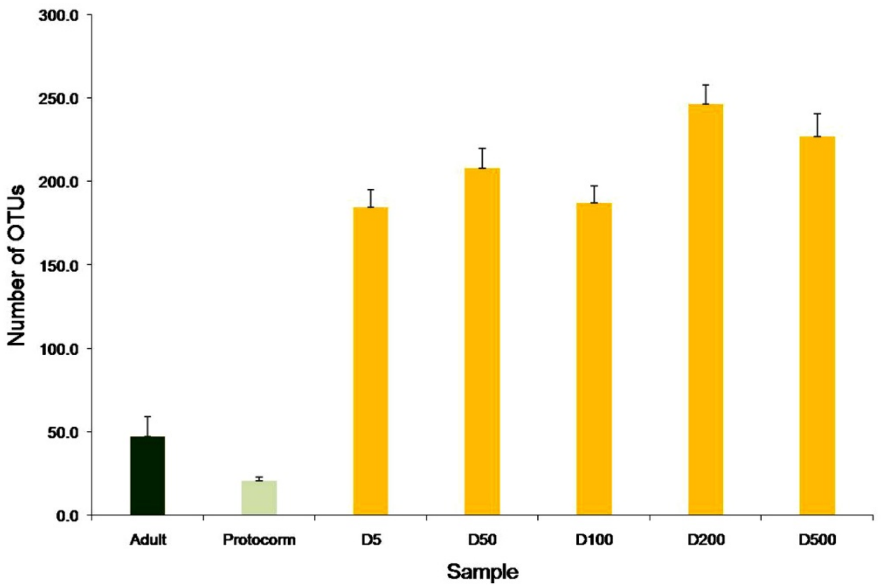

Fig. S5 Histogram of fungal communities in the surrounding litter samples at three taxonomic levels (A) Class level, (B) Order level and (C) Family level. At each level, we selected the 10 most abundant taxonomies (only the top 5 at the class level) based on the mean percentage across all samples.

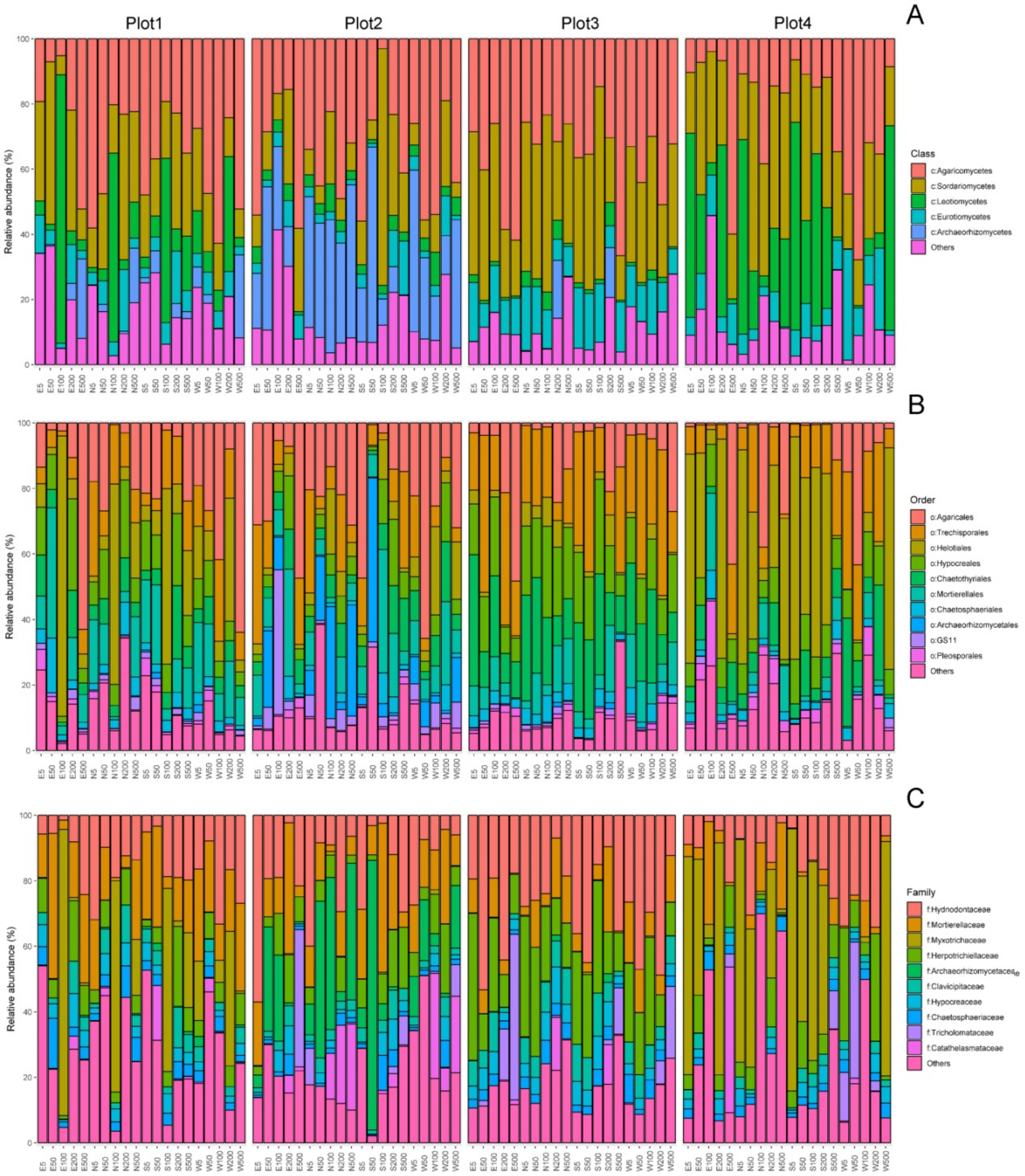

Fig. S6 Non-metric multidimensional scaling (NMDS) plot illustrating differences in fungal communities in soil samples collected at four different plots within bamboo forest. Each data point represents an individual soil sample.

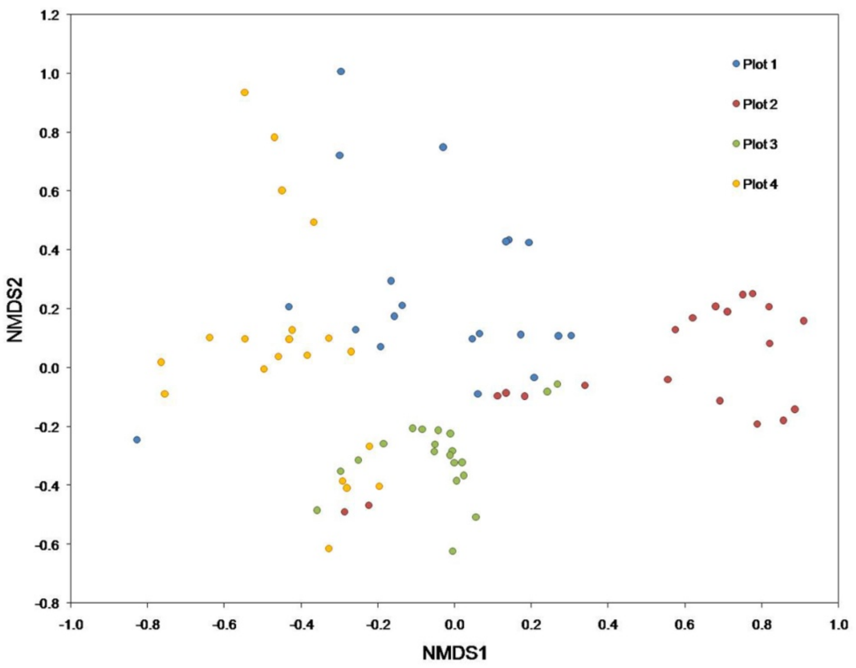

Fig. S7 Phylogenetic relationships of mycorrhizal fungi of *Gastrodia confusoides* (OTU3 and OTU5) using ITS1 region sequences of *Mycena* and *Gymnopus* available in GenBank. Phylogenetic analysis was conducted using neighbor-joining with 1000 bootstrap replicates (values of more than 70% are above the branches). *Cladosporium* isolate was used as an outgroup.

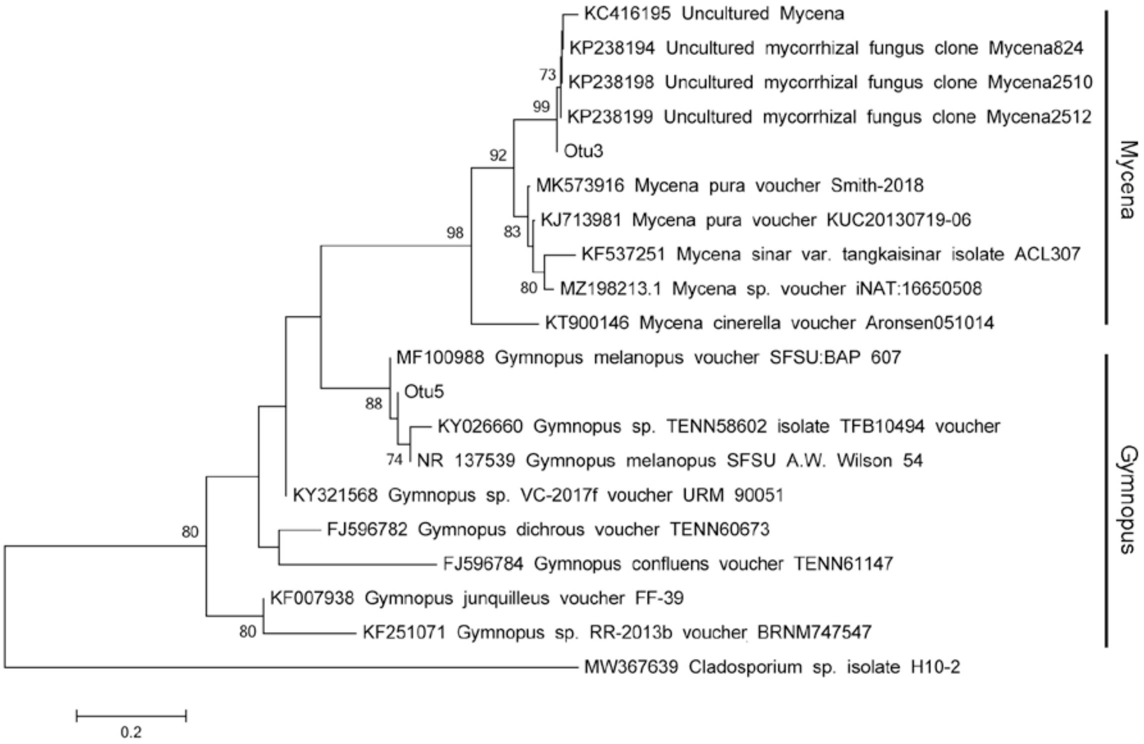

Fig. S8 For *Mycena* (OTU3) and *Gymnopus* (OTU5), no significant relationship between the distance from the orchid plant and fungal abundance determined by qPCR was observed in bamboo litter surrounding the orchid plant ($\chi^2 = 4.40$ and 6.39, $p > 0.05$ for OTU3 and OTU5, respectively).

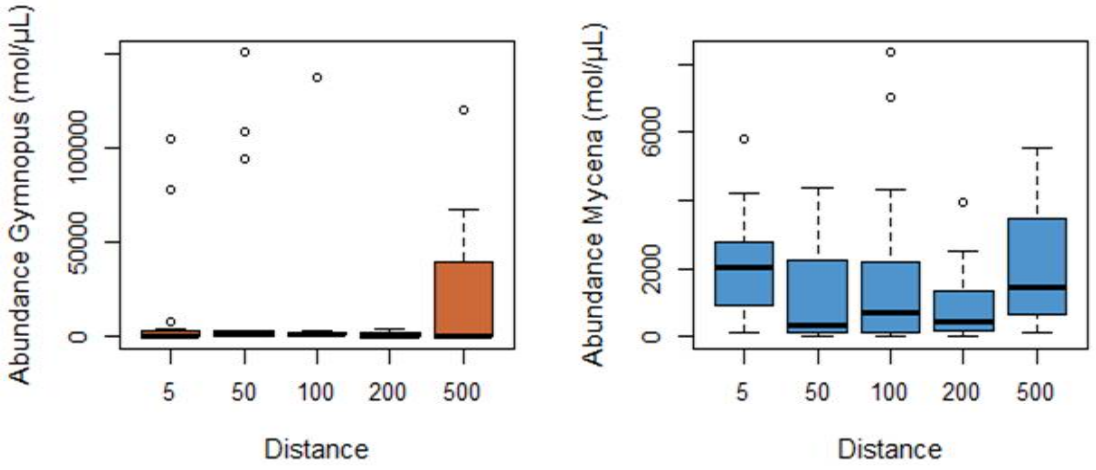

Supplement: Supplementary file 1 [file Data_Sheet_1.zip › Supplementary Material/Supplementary Tables and Figures.PDF]
